# Clinical feasibility of combining intraoperative electron radiation therapy with minimally invasive surgery: a potential for electron-FLASH clinical development

**DOI:** 10.1007/s12094-022-02955-z

**Published:** 2022-09-28

**Authors:** Felipe Ángel Calvo Manuel, Javier Serrano, Claudio Solé, Mauricio Cambeiro, Jacobo Palma, Javier Aristu, Jose Luis Garcia-Sabrido, Miguel Angel Cuesta, Emilio del Valle, Fernando Lapuente, Bernardino Miñana, Miguel Ángel Morcillo, Jose Manuel Asencio, Javier Pascau

**Affiliations:** 1 Department of Oncology, Clinica Universidad de Navarra, Madrid-Pamplona, Spain; 2 Instituto RadioMedicina, Santiago del Chile, Chile; 3 Hospital General Universitario Gregorio Marañon, Madrid, Spain; 4 Amsterdam University Medical Center, Holland, Netherlands; 5 Department of Surgery, Clinica Universidad de Navarra, Madrid, Spain; 6 Department of Urology, Clinica Universidad de Navarra, Madrid, Spain; 7 Radiobiology Division, CIEMAT, Madrid, Spain; 8 Department of Bioengineering and Aerospace Engineering, Universidad Carlos III de Madrid, Getafe, Spain

**Keywords:** Intraoperative radiation therapy, Electron beams, Laparoscopic surgery, Robotic surgery, Cancer surgery, FLASH

## Abstract

**Background:**

Local cancer therapy by combining real-time surgical exploration and resection with delivery of a single dose of high-energy electron irradiation entails a very precise and effective local therapeutic approach. Integrating the benefits from minimally invasive surgical techniques with the very precise delivery of intraoperative electron irradiation results in an efficient combined modality therapy.

**Methods:**

Patients with locally advanced disease, who are candidates for laparoscopic and/or thoracoscopic surgery, received an integrated multimodal management. Preoperative treatment included induction chemotherapy and/or chemoradiation, followed by laparoscopic surgery and intraoperative electron radiation therapy.

**Results:**

In a period of 5 consecutive years, 125 rectal cancer patients were treated, of which 35% underwent a laparoscopic approach. We found no differences in cancer outcomes and tolerance between the open and laparoscopic groups. Two esophageal cancer patients were treated with IOeRT during thoracoscopic resection, with the resection specimens showing intense downstaging effects. Two oligo-recurrent prostatic cancer patients (isolated nodal progression) had a robotic-assisted surgical resection and post-lymphadenectomy electron boost on the vascular and lateral pelvic wall.

**Conclusions:**

Minimally invasive and robotic-assisted surgery is feasible to combine with intraoperative electron radiation therapy and offers a new model explored with electron-FLASH beams.

## Introduction

### Intraoperative electron radiation therapy (IOeRT) and laparoscopic surgery: the locally advanced rectal cancer model

Laparoscopic resection is a surgical standard: IOeRT is feasible to be integrated to boost post-resection pelvic areas at high risk.

In experienced groups, oncologic surgery (e.g., lymph node removal, negative resection margins) is performed using laparoscopy [1]. In patients with rectal cancer, laparoscopy-assisted surgical resection is safe after preoperative CRT and the quality of resection is equivalent to that obtained using an open procedure [2]. Although laparoscopic surgery has the advantages of reduced postoperative ileus and pain and a shorter length of stay than open surgery [2], the literature contains few reports of laparoscopic IOeRT for rectal cancer [3, 4].

The positive experience with laparoscopic surgery to early-stage rectal cancer led us to incorporate this approach in the multimodal management of locally advanced rectal cancer (LARC) patients. Preoperative treatment including induction chemotherapy and chemoradiation was followed by laparoscopic surgery and IOeRT delivered to the posterior pelvic wall (post-resection and pre-reconstruction).

## Materials and methods

Methodology and patient selection: single-institution IOeRT expert experience.

**Fig. 1:**
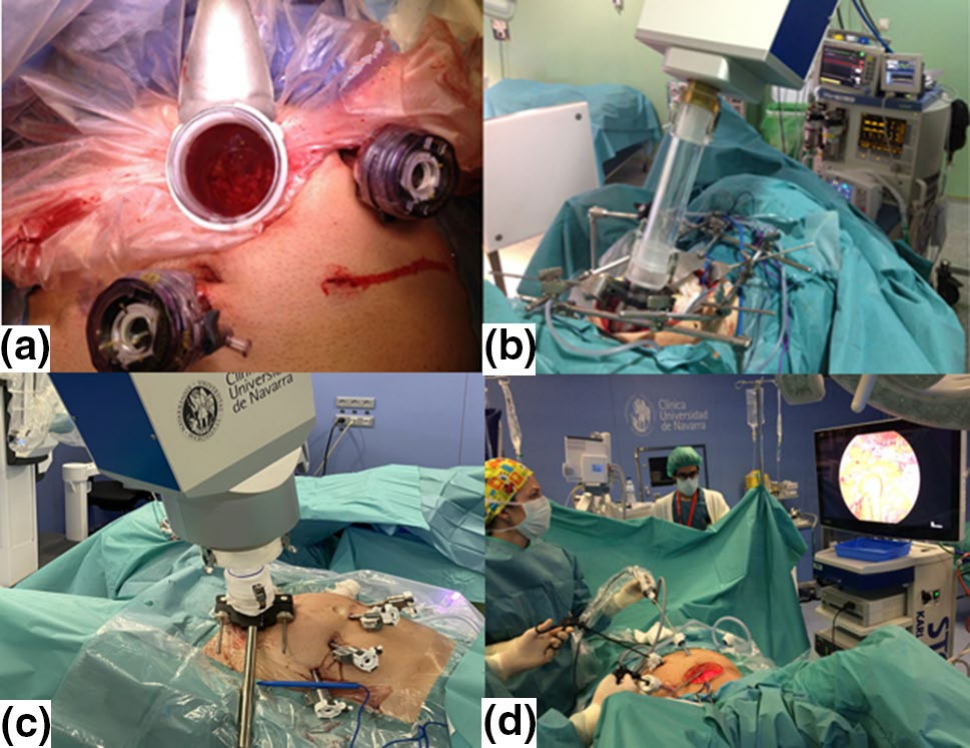
IOeRT procedure for rectal cancer: **a** Laparoscopic approach: the retractor removes the rectal stump and the anterior pelvic structures out of the electron beam applicator; **b** open procedure vision: retractors in place to assure presacral space exposure with protection of pelvic sensitive structures and organs; **c** suprapubic laparotomy incision to extract the resected specimen and introduce the electron applicator during a laparoscopic procedure and docking completed to the linear accelerator; **d** internal visual control of the tumor bed after applicator positioning

In the period June 2005 to December 2010, 125 patients with LARC [cT3-T4 and/or cN + , staged with endoscopic ultrasound and with pelvic magnetic resonance imaging] met these criteria [5]. A total of 12 senior surgeons (3 surgical teams) were involved in the program. The process of assignment to a surgical team (laparoscopic or open surgery) was done by clinical condition (e.g., extreme obesity precluded laparoscopic surgery). Forty-four patients (35%) were treated according to the laparoscopic protocol. The remaining 81 patients (65%) were treated with an open surgical approach and served as the control cohort (retrospective case–control study). All patients received two courses of induction FOLFOX-4 (oxaliplatin, leucovorin, 5-fluorouracil) [6]. Thereafter, preoperative conformal three-dimensional radiation therapy was used to deliver a tumor dose of 50.4 Gy with concurrent oral tegafur (oral 5-FU prodrug). Adjuvant chemotherapy (4–6 courses) consisting of a bolus of 5-fluorouracil (425 mg/m^2^) combined with leucovorin (20 mg/m^2^) on days 1–5 every 21 days was recommended following the institutional protocol. Radical surgery was scheduled for 6 weeks after completion of CRT. Compliance with the principles of TME was mandatory. After surgery and before pelvic reconstruction, 10–15 Gy (median 12.5 Gy) was delivered in a single fraction (via suprapubically localized mini-laparotomy [Pfannenstiel incision]) to the posterior presacral space, using a median energy of 12 MeV (Fig. 1). The dose was determined based on the completeness of surgical resection, considering the possibility of a positive radial resection margin. Posttreatment changes observed in rectal cancer specimens were staged according to the sixth edition of the AJCC classification (ypTNM) [7].

## Results

### Procedures-related radiosurgical results: mature cancer outcomes

Table 1 presents the demographics and the tumor-, treatment-, and outcome-related data of the cohorts treated with laparoscopic or open surgery in the same time period. No statistically significant differences were found in any of the relevant clinical–therapeutic parameters analyzed, except for more female patients in the laparoscopy group (59 vs. 33%, *p* = 0.005).

**Table 1:** Demographics and outcomes in an IOeRT expert institution experience on rectal cancer (2005–2010)

Variable	Total [*n* = 125(%)]	Laparoscopic surgery [*n* = 44 (35%)]	Open surgery [*n* = 81 (65%)]	*P* value
Demographics				
Median age (range)	63 (31–86)	62 (31–84)	64 (33–86)	0.62
Male	72 (58)	18 (41)	54 (67)	0.005
Female	53 (42)	26 (59)	27 (33)	
Median time interval to surgery (days)	47 (22–83)	46 (22–83)	47 (25–78)	0.83
Location of tumor (distance to anal verge)				
Lower rectum (< 5 cm)	41 (33)	15 (34)	26 (32)	0.93
Clinical tumor stage				
cT2–T3	93 (74)	30 (68)	63 (79)	0.24
cT4	32 (26)	14 (32)	18 (22)	
Clinical nodal stage				
cN0	12 (10)	2 (5)	10 (12)	0.16
cN +	113 (90)	42 (95)	71 (88)	
Median IOeRT cone size	6 (5–8)	6 (5–8)	6 (5–8)	0.92
Median IOeRT dose (cGy)	1.250 (1.000–1.500)	1.250 (1.000–1.500)	1.250 (1.000–1.500)	0.94
Median IOeRT energy (MeV)	12 (6–18)	12 (6–18)	12 (6–18)	0.93
Outcomes				
Intervention				
Abdominoperineal	36 (29)	15 (34)	21 (26)	0.5
Anterior resection	8 (6)	2 (5)	6 (7)	
Low anterior resection	52 (42)	20 (45)	32 (40)	
Ultralow anterior resection	29 (23)	7 (16)	22 (27)	
Total mesorectal excision				
Complete	119 (95)	43 (98)	76 (94)	0.44
Incomplete	6 (5)	1 (2)	5 (6)	
Pathologic tumor stage				
ypT3	49 (39)	15 (34)	34 (42)	
ypT4	7 (6)	07	7 (8.5)	
Pathologic nodal stage				
ypN0	90 (72)	32 (73)	58 (71)	

Patients in the laparoscopy group lost less blood and had a shorter hospital stay than those in the open surgery group. Postoperative complications (at least 1 postoperative complication) were reported in 14 of 44 patients (32%) in the laparoscopy group and in 36 of 81 patients (44%) in the open surgery group. The proportion of patients who underwent a second intervention within 28 days after surgery was similar between the two groups. Intraoperative, acute, and late toxicity was similar among groups.

The distribution of the different procedures was similar in both groups. An abdominoperineal procedure was performed in 34% of the patients in the laparoscopy group and in 26% in the open surgery group. Laparoscopic procedures were converted to open surgery in 5 of 44 patients. Macroscopically incomplete resected specimens were obtained in 1 of 44 patients (2%) after laparoscopic surgery and 5 of 81 (6%) after open surgery. The median distal resection margin was 2.84 cm in the laparoscopic group and 3.17 cm in the open surgery group (*p* = 0.25). The median distance to the circumferential resection margin was 7 cm in the laparoscopic group and 8 mm in the open surgery group (*p* = 0.42). The median number of lymph nodes harvested after surgery was not significantly different between the groups. Tumor and nodal downstaging after preoperative treatment did not differ significantly between the two groups. Median follow-up time for the entire cohort of patients was 59.5 months (range 7.8–90). Seven patients had a loco-regional recurrence (5.6%), and 22 out of the original 125 patients (18%) developed distant metastases. Two out of the seven patients with loco-regional recurrence were rescued with a second surgical procedure and achieved long-term survival (24 and 55 months).

**Fig. 2:**
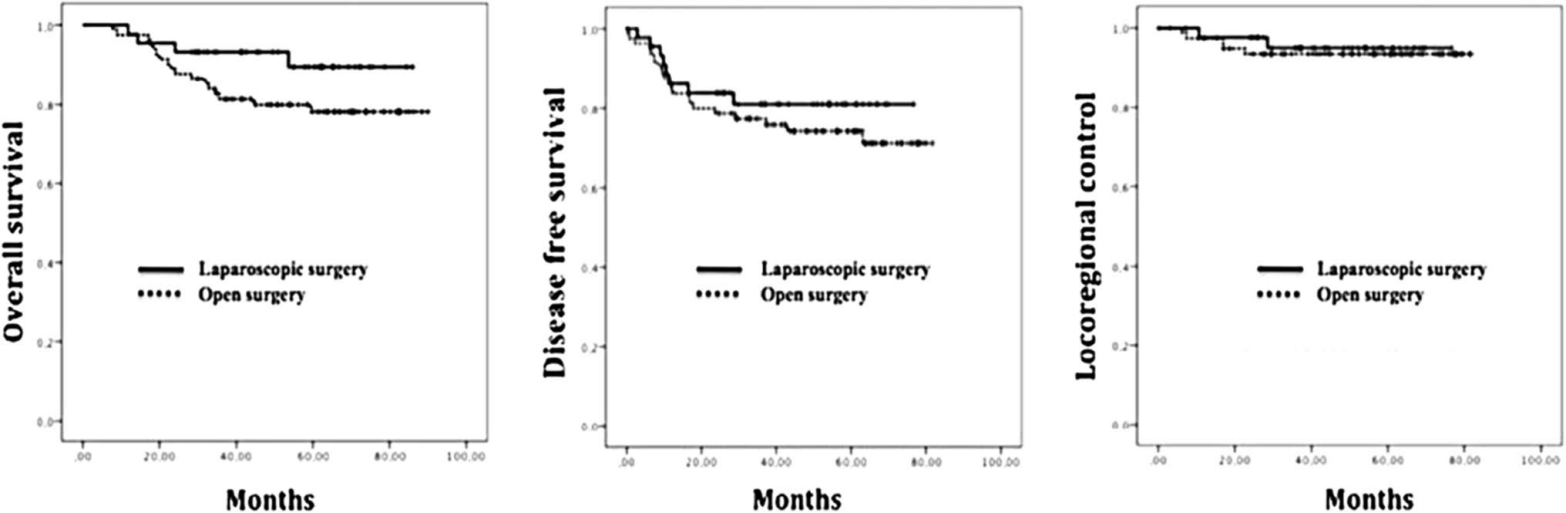
Rectal cancer outcomes in patients treated in an expert IOeRT institution comparing laparoscopy or open surgical approaches after preoperative combined modality therapy

No significant differences in 5-year OS were detected between the groups (Fig. 2). Disease-free survival at 5 years was 74.1%. Multivariate analysis showed that distal margin ≤ 10 mm, CRM ≤ 1 mm, tumor grade 3, tumor regression grade 3–4, and ypN + disease were significantly associated with disease-free survival. Five-year LRC was 94% (Fig. 1c). No significant differences in 5-year loco-regional control were observed between the laparoscopic and the open surgery groups (Fig. 2). Multivariate analysis showed that distal margin ≤ 10 mm, CRM ≤ 1 mm, and tumor grade 3 were statistically associated with loco-regional control.

## Discussion

### The evolution of laparoscopy and IOeRT in the management of rectal cancer patients

The results of several prospective randomized trials on laparoscopic resection for mid- to low rectal cancer have been reported [1, 2, 8–10]. A large randomized trial (1103 patients) showed less blood loss in the laparoscopy group compared to the open surgery group (median 200 vs 400 ml, *p* < 0.0001). However, laparoscopy prolonged surgical times (240 vs 188 min; *p* < 0.0001). In the laparoscopy group, bowel function was regained sooner (2.0 vs. 3.0 days; *p* < 0.0001) and hospital stay was shorter (8.0 vs. 9.0 days; *p* = 0.036). Macroscopically, the completeness of resection was not different between the groups (88 vs 92%; *p* = 0.250). A positive CRM (< 2 mm) was noted in 10% of patients in both the laparoscopy and the open surgery groups (*p* = 0.850). Postoperative morbidity (40% vs 37%; *p* = 0.424) and mortality (1% vs 2%, *p* = 0.409) within 28 days after surgery were similar.

A recent meta-analysis (7 randomized trials involving 1767 laparoscopic and 1293 open resections for rectal cancer) concluded that laparoscopic rectal cancer resection does not compromise long-term oncologic outcomes compared with open surgery with potential survival benefits (superior disease-free survival) for a minimal access approach in patients with stage II and III rectal cancer [11].

An important remark is that most of the laparoscopic data were generated excluding patients with T3 rectal cancer within 2 mm of the endopelvic fascia or T4 cancers. Therefore, their findings require validation in LARC patients after contemporary multidisciplinary treatment including exposure to intensive preoperative systemic and radiation treatments. The demonstration of the equivalence and possibly benefits in some of the parameters analyzing clinical tolerance and the induction of similar downstaging effects, without increased toxicity in tissues exposed to extensive combined modality therapy and different surgical approaches, will form solid evidence to promote the use of minimally invasive surgery and IOeRT also in patients with locally advanced disease at diagnosis.

Most of the challenges in the treatment of rectal cancer concern the lowest located and the locally advanced tumors. Several reports have described the availability and effectiveness of IOeRT combined with preoperative chemoradiation for controlling LARC [12] and promoting improved local control results compared to non-IOeRT strategies based on systematic reviews [13, 14]. In particular, the data reported in cT4 patients and isolated recurrences, using IOeRT as a component of local intensification treatments [15, 16], are incorporated in the experts’ guidelines as a component of the multimodal treatment that is recommended in clinical practice [17, 18]. Laparoscopic dissection preceded by a multimodal preoperative treatment combines accuracy with reduced invasiveness and faster recovery. Long-term outcomes are encouraging, and confirmatory larger series are necessary to draw a comparison with the long-term outcomes of open surgery so that laparoscopy can be further incorporated into the radiosurgical practice.

Locally advanced rectal cancer has broadly been defined as T3, T4, or lymph node-positive disease. Preoperative chemoradiation is the standard of care, based on acceptable toxicity and reduced local recurrence rates, as well as higher rates of sphincter preservation compared with postoperative chemoradiation. Both short-course radiation and long-course chemoradiation followed by TME and adjuvant chemotherapy are currently accepted methods, with recent trials showing equivalence in outcomes, with longer follow-up ongoing. MRI is increasingly being used to determine pCR following preoperative therapy to either predict optimal surgical candidates or as a mechanism for monitoring patients without immediate surgical intervention. Currently, TME remains the cornerstone of treatment for locally advanced rectal cancer, nonoperative management is an emerging alternative treatment paradigm for achieving comparable oncologic control with encouraging early results [19].

### Intraoperative electron radiation therapy (IOeRT) and minimally invasive surgery: the esophageal cancer model

Outcomes including intraoperative electron irradiation in the multimodal approach have been reported in the treatment of locally advanced gastric cancer [20–22], gastroesophageal [23] and esophageal cancer [24]. High loco-regional control rates and tolerability in dose-escalated trials designed were described. Local control promotion was observed using IOeRT as a boost (combined with external conventional irradiation) and this effect was significant compared to surgery and external irradiation standard combination [24] or to the surgery alone approach [25]. The results observed in the esophageal cancer model to the combination of surgery plus external irradiation and electron boost after esophagectomy and mediastinal dissection have reported favorable tolerance with nerve-spacing maneuvers and multiple field arrangements [26]. Additionally, promotion of loco-regional control by boosting the upper abdominal lymphatic regions with IOeRT has been described [27, 28].

### Intraoperative electron radiation therapy (IOeRT) in esophago-gastric cancer and open surgery: institutional experiences and meta-analysis

A meta-analysis including 11 studies, 9 for gastric cancer and 2 for esophageal cancer reported [29] on 1581 patients, 570 in the IOeRT group and 1011 in the control group. IOeRT showed favorable effects for patients with cancer in stage II and stage III with improvement of loco-regional control. Complications were similar between the IOeRT group and control group (OR = 1.15; 95% CI 0.77–1.72; *P* = 0.50). The results supported findings observed in a previous meta-analysis showing a statistically significant loco-regional control benefit with the addition of IOeRT in patients with resectable gastric cancer and the indication that IOeRT may provide promising results on overall survival for the subgroup of patients with stage III disease [30].

**Fig. 3:**
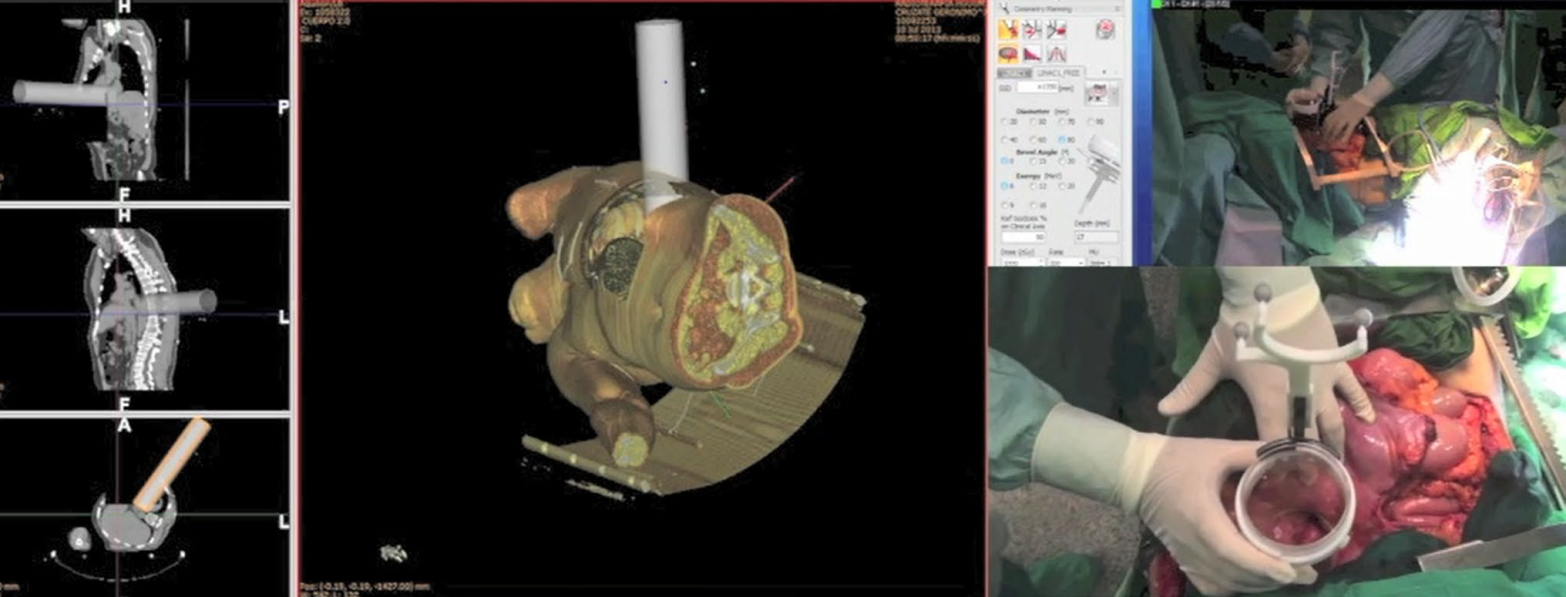
Intraoperative electron irradiation (IOeRT) during open surgery for gastro-esophageal cancer. Lateral thoracotomy to approach circumferential resection margin in the lower mediastinum and nodal regions in the upper abdomen. 3D reconstruction of the anatomy and applicator positioning

Results from a prospective registry [31] (Hospital General Universitario Gregorio Marañon, Madrid, Spain) of patients treated with and without IOeRT were reported. The radiation boost was integrated in an intensive multimodality treatment approach. A retrospective analysis reported feasibility, tolerance, anatomical topography of loco-regional recurrence, and long-term outcome for esophageal and esophago-gastric cancer patients treated with preoperative chemoradiation and surgery with or without a radiation boost of IOeRT to the areas at risk or involved after resection (Fig. 1). In summary, 53 patients with primary esophageal (*n* = 26; 44%) or esophago-gastric carcinoma (*n* = 30; 56%), with disease confined to loco-regional area, were evaluated. Thirty-seven patients also received a perianastomotic reconstruction IOeRT boost over the tumor bed in the mediastinum and upper abdominal lymph node area. Loco-regional recurrence rate was 15% (*n* = 8). Five-year overall survival and disease-free survival was 48 and 36%, respectively. Univariate and multivariate log-rank analyses showed that receiving IOeRT was associated with lower risk of local recurrence (*p* = 0.004; *p* = 0.01) (Fig. 3).

### Esophagectomy and minimally invasive surgery in cancer patients

Overall progress in the treatment of local and locally advanced esophageal, gastroesophageal junction, and gastric adenocarcinomas led to level-one evidence including heterogeneous histologies and locations, associated with these tumors. The accepted standard of chemoradiotherapy for locally advanced esophageal and gastroesophageal junction cancers is based on trials. However, staging evaluations are not uniform and optimal chemotherapy and total dose of radiation remain controversial [32]. Innovative technologies for minimally invasive surgery for esophageal resection [33, 34] have proved the feasibility and safety, and several systematic review and meta-analysis have identified significant clinical benefits in outcomes for minimally open approaches versus traditional open techniques [35, 36]. Esophagectomy for cancer is associated with a high risk of complications. A minimally invasive approach might be less traumatic, leading to fewer complications and may also improve the oncological outcome. The meta-analysis of six RCTs including 822 patients showed a lower risk of postoperative complications compared to open resection. Overall and disease-free survival was comparable for the two techniques [37].

Clinical experiences during mentoring and training of minimally invasive esophagectomy and the feasibility of intraoperative electron radiation therapy were explored (IOeRT).

**Fig. 4:**
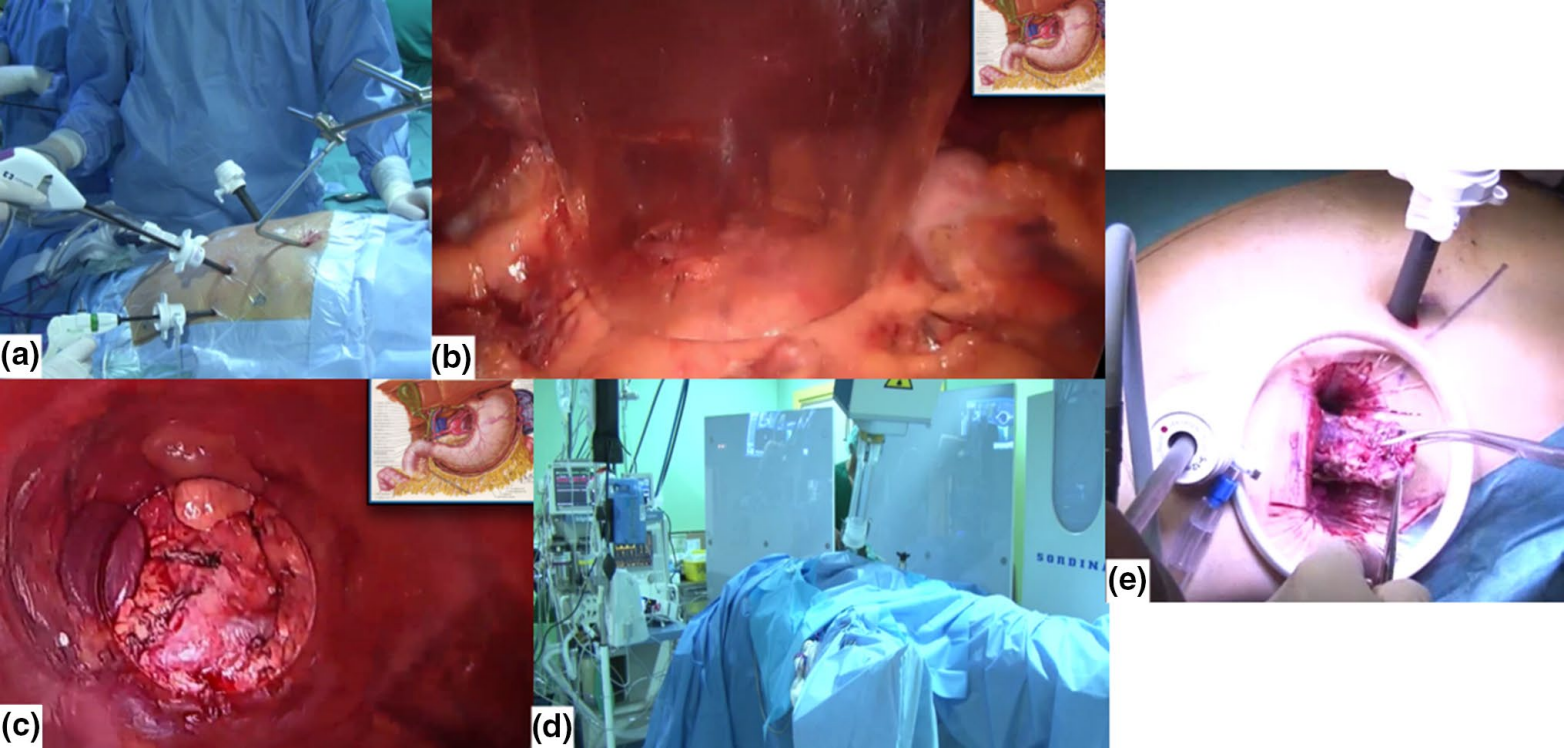
View of minimally invasive esophagectomy procedure and intraoperative electron irradiation. **a** Abdominal time: laparoscopic approach; **b** intraabdominal view of celiac trunk boost after nodal resection. Notice that the stomach, colon, small bowel, duodenum and hepatic hilum are displaced and protected from the electron beam directed by the applicator; **c** beam’s-eye view of the selected target at the celiac trunk; **d** miniaturized linear accelerator after the docking procedure delivering the radiation beam; **e** thoracoscopic time and extraction of the final surgical specimen

Since the first laparoscopic procedure, there has been a steady increase in advanced minimally invasive surgery. These procedures include oncological colorectal, hepatobiliary, and upper gastrointestinal surgery. Implementation of these procedures requires different and new skills for the surgeons who wish to perform these procedures. To accomplish this surgical teaching program, a mentorship seems the most ideal method to teach the apprentice surgeon these specific skills. A pioneering teaching program for a minimally invasive esophagectomy for esophageal cancer was started in 2009 [38]. As part of the mentoring cancer institutes throughout Europe, the Hospital General Universitario Gregorio Marañon (Madrid, Spain) explored the feasibility of integrating of intraoperative electron irradiation while the mentoring process in the Surgical Department by Prof. Cuesta as part of the innovation launched for esophageal cancer patients requiring multimodal therapy for locally advanced cancer stages [31] (Fig. 4). Patients piloting this experience are described in Table 2.

**Table 2:** Patients treated with thoracoscopic esophagectomy and intraoperative electron irradiation following neoadjuvant therapy

Variables	Case 1	Case 2
Age	72	50
Gender	Male	Female
Co-morbidity	Dyslipidemia, pericarditis	–
Cancer	Esophageal	Esophageal
Location	Esophago-gastric junction	Esophago-gastric junction
Histology	Adenocarcinoma	Adenocarcinoma
Stage	cT2N1Mx	
Status	Primary locally advanced	Primary locally advanced
Previous Tx	Chemoradiation 45 Gy +	Chemoradiation 45 Gy +
Response	Minor endoscopic	Major endoscopic
Surgery		
Duration	8 h 10 min	9 h 15 min
IOeRT		
Applicator size	5 cm	5 cm
Beveled angle	30°	30°
Dose	15 Gy	15 Gy
Energy	10 MeV	12 MeV
Target	Celiac trunk	Celiac trunk
Access	Abdominal	Abdominal
Pathology	YpT2 YpN2	YpT0 YpN0
Hospital discharge	5 days	12 days
Outcomes		
Loco-regional control	18 months	48 months

### Intraoperative electron radiation therapy and robotic-assisted surgery: initial clinical experiences

**Fig. 5:**
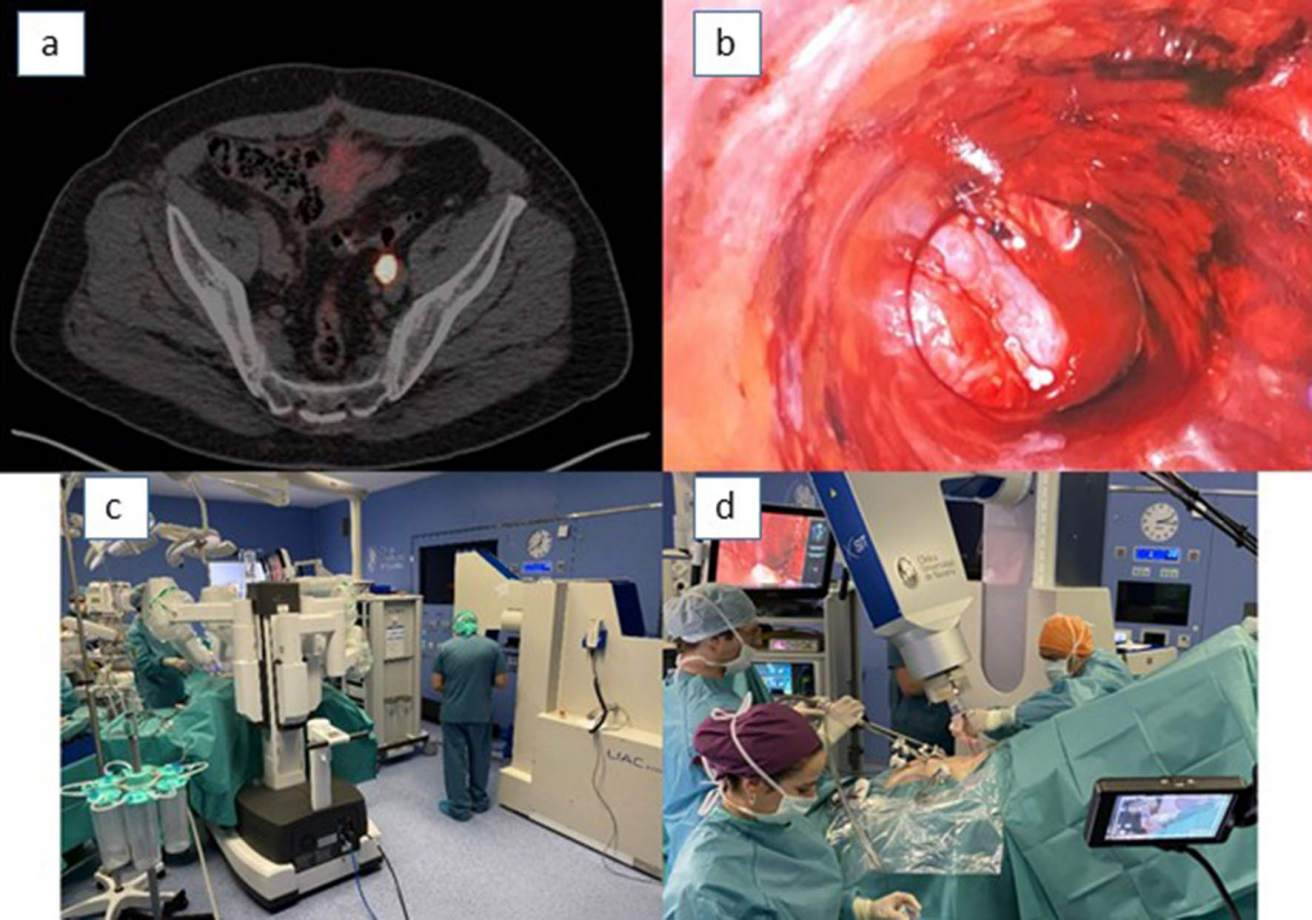
View of the IOeRT procedure during robotic surgery. **a** PET-PSMA showing a single nodal recurrence at the common iliac vessels, 2.7 cm in maximal diameter; **b** after nodal removal, the superior and inferior limit of the nodal bed is marked with fiducials seen via the interior of the applicator (4 cm in diameter), while intrapelvic normal tissues uninvolved by cancer are displaced from the electron beam (ureter, bladder, rectum, small bowel); **c** operating room view with miniaturized technologies present at the time of the surgical procedure: left da Vinci robotic system, right IOeRT linear accelerator; **d** docking procedure sequential to robotic resection, and IOeRT applicator positioning under video control while the pneumoperitoneum is maintained

The theoretical advantages of robotic surgery are the enhanced three-dimensional and magnified views of closed spaces that can be obtained as well as increased dexterity with fine precision surgical instruments, which make it ideal for operating in confined anatomical spaces such as the pelvis. Considering the great complexity of pelvic anatomy, robotic surgery has been reported to be feasible and related to beneficial outcomes in urological malignancy including prostatectomy [39] and nodal dissections [40], rectal [41], and exenterative pelvic surgery [42]. Recently, robotic esophagectomy is reported as an extension of this technology to the thoracic cavity [43–45]. Table 3 describes the demographics and characteristics of tumor and treatment involved in the initial experience at Clinica Universidad de Navarra in the rescue of oligo-recurrent patients with prostate cancer diagnosis (Fig. 5).

**Table 3:** Patients treated with initial clinical experience integrating electron intraoperative irradiation (IOeRT) with robotic-assisted surgical nodal resection

Variables	Case 1	Case 2
Age (years old)	62	75
Cancer	Prostate	Prostate
Histology	Adenocarcinoma	Adenocarcinoma
Gleason	3 + 3	4 + 3
Stage	Recurrent	Recurrent
Time to rescue	7 years	11 years post-prostatectomy
		7 years post-radiotherapy
Status	Localized nodal relapse	Localized nodal relapse
Previous therapy	IMRT 66 Gy	IMRT 60 Gy pelvic field
	HT 3 years	HT 1 year
PET-PSMA	SUV 19.5	14.7
Disease site	Ilio-obturator	common iliac
Disease size	2.4 cm	2.7 cm
PSA at rescue	2.27 ng/ml	3.13 ng/ml
Type of resection	Lymphadenectomy pelvic	Lymphadenectomy pelvic
IOeRT		
Applicator size	5 cm	4 cm
Beveled end	30°	30°
Energy	8 MeV	12 MeV
Dose	15 Gy	15 Gy
Surgical time	4 h 33 min	3 h 45 min
Incidences	–	–
Pathology	Extracapsular extension	Extracapsular extension
Follow-up		
	Biochemical remission	Biochemical remission
	8 months	18 months

*HT* hormonal therapy, *IMRT* intensity-modulated radiation therapy

### The potential of intraoperative electron-FLASH and laparoscopic or thoracoscopic cancer resection

FLASH irradiation with electron beams has proven to be protective in normal tissue damage in animal models, while maintaining similar tumor control effect [46]. Electron-FLASH technology allows integration into current IOeRT strategies, similar to the successful investigations in human cancer models [47]. Minimally invasive laparoscopic or thoracoscopic surgery, as a relevant progress in cancer surgery, can be integrated with intraoperative high-energy electron irradiation. The IOeRT dose can adequately be directed at the predefined high-risk cancer target area by modifying the incision used to resect and extract the surgical specimen.

IOeRT can be delivered as a boost component after preoperative chemoradiation in gastroesophageal cancer as a dose-escalation strategy to improve local tumor control in the nodal regions of the upper abdomen (laparoscopic modality) and in the lower mediastinal space covering the circumferential margin tumor extension (thoracoscopic approach). The normal tissues exposed to electron boost are relatively dose resistant (vessels, pancreas, aorta, prevertebral ligament and vertebral body, which remains however limited due to the single dose concept of IOeRT [48–52]. In the pelvic region, after radical rectal cancer resection following preoperative chemoradiation, dose-sensitive tissues include the peripheral nerve structures in the presacral space and in the lateral pelvic wall regions. Peripheral neuropathy is a well-described toxicity related to the use of escalated IOeRT [53].

A promising relevant potential of using electron-FLASH for IOeRT dose delivery entails the sparing of normal tissue effects, lowering the risks for late side effects while maintaining tumor control or, in the case of escalation of cancer dose, even further improving disease-related outcome parameters. Another scenario of particular interest to explore electron-FLASH IOeRT combined with surgery, in this case after reducing or omitting the preoperative external beam radiation therapy component, concerns oligo-recurrent cancer patients in intrapelvic sites that are previously irradiated and/or already symptomatic due to direct cancer involvement [54].

Laparoscopic liver resection is an alternative approach to open surgery for hepatocellular carcinoma and metastatic liver disease [55]. Liver tolerance in large animal models to intraoperative electrons has a favorable profile and is an additional area of research and development for the electron-FLASH technology [56–59].

### The potential of electron-FLASH in cancer patients who are candidates for robotic surgery

Robotic surgery for cancer patients is well established, e.g., for prostatectomy, pelvic nodal resection and a number of sites of oligo-metastatic or -recurrent disease. IOeRT has been explored pre-prostatectomy, combining open surgery with 10–12 Gy of IOeRT delivered with 9–12 MeV electron energy beams. In vivo dosimetry was feasible and accurate in terms of dose deposit prediction [60]. A similar approach integrating the delivery of 12 Gy IOeRT after exposure of the prostate, followed by prostatectomy, compared to prostatectomy with or without external beam, and irradiation if indicated (matched pair analysis) did not show differences in continence rate and no major complications in either group. The acute and late toxicity and biochemical progression-free survival were equivalent [61]. The tolerance of dose-sensitive tissues to high-single doses of electrons as delivered intraoperatively have been studied in large animal models including the ureter [62], bladder [53, 63], and the peripheral nerves. The studies described that below 15 Gy, detectable histopathological tissue damage or functional impairment was not observed. Electron-FLASH offers now the opportunity to further explore increased doses of IOeRT with robotic prostatectomy to further improve local control, while minimally compromising continence and erectile function.

## Conclusions

State-of-the-art cancer care through medical innovation opens a significant opportunity for individualizing cancer management across a broad spectrum of diseases. Minimally invasive surgery significantly improves the tolerance for surgical procedures in cancer patients, while maintaining established quality standards in cancer surgery. The combination of improved surgical standards and improved delivery of radiation therapy forms an essential component of integral quality oncologic care. Intraoperative electron radiation therapy delivery is feasible during laparoscopic, thoracoscopic, and robotic-assisted surgical procedures. An electron beam delivered at ultrahigh dose rates (electron-FLASH) promises to further improve the tolerance of normal tissues. In this scenario, escalating the intraoperative dose delivered during minimally invasive surgery might significantly improve local tumor control without jeopardizing normal tissue tolerance.

## References

[1] Kang S-B, Park JW, Jeong S-Y, Nam BH, Choi HS, Kim D-W (2010). Open versus laparoscopic surgery for mid or low rectal cancer after neoadjuvant chemoradiotherapy (COREAN trial): short-term outcomes of an open-label randomised controlled trial. Lancet Oncol.

[2] van der Pas MH, Haglind E, Cuesta MA, Fürst A, Lacy AM, Hop WC (2013). Laparoscopic versus open surgery for rectal cancer (COLOR II): short-term outcomes of a randomised, phase 3 trial. Lancet Oncol.

[3] Civello IM, Cavicchioni C, Tacchino RM, Matera D, Valentini V, Manfrida S (2007). Laparoscopic resection with intraoperative radiotherapy: a new step in the multimodal treatment of advanced colorectal cancer. Surg Endosc.

[4] Skrovina M, Soumarova R, Duda M, Bezdek R, Bartos J, Wendrinski A (2014). Laparoscopic abdominoperineal resection with intraoperative radiotherapy for locally advanced low rectal cancer. Biomed Pap Med Fac Univ Palacky Olomouc Czech Repub.

[5] Calvo FA, Sole CV, Serrano J, Rodriguez M, Marcos F, Muñoz-Calero A (2013). Postchemoradiation laparoscopic resection and intraoperative electron-beam radiation boost in locally advanced rectal cancer: long-term outcomes. J Cancer Res Clin Oncol.

[6] Calvo FA, Serrano FJ, Diaz-González JA, Gomez-Espi M, Lozano E, Garcia R (2006). Improved incidence of pT0 downstaged surgical specimens in locally advanced rectal cancer (LARC) treated with induction oxaliplatin plus 5-fluorouracil and preoperative chemoradiation. Ann Oncol.

[7] Edge SB, Byrd DR, Compton CC, Fritz AG, Greene FL, Trotti A, American Joint Committee on Cancer (2007). General information on cancer staging and end-results reporting. Cancer staging handbook.

[8] Green BL, Marshall HC, Collinson F, Quirke P, Guillou P, Jayne DG (2013). Long-term follow-up of the Medical Research Council CLASICC trial of conventional versus laparoscopically assisted resection in colorectal cancer. Br J Surg.

[9] Jayne DG, Guillou PJ, Thorpe H, Quirke P, Copeland J, Smith AMH (2007). Randomized trial of laparoscopic-assisted resection of colorectal carcinoma: 3-year results of the UK MRC CLASICC Trial Group. J Clin Oncol.

[10] Ng SSM, Lee JFY, Yiu RYC, Li JCM, Hon SSF, Mak TWC (2014). Laparoscopic-assisted versus open total mesorectal excision with anal sphincter preservation for mid and low rectal cancer: a prospective, randomized trial. Surg Endosc.

[11] Lim WH, Tan DJH, Ng CH, Syn N, Tai BC, Gu T (2021). Laparoscopic versus open resection for rectal cancer: an individual patient data meta analysis of randomized controlled trials. Eur J Surg Oncol.

[12] Sole CV, Calvo FA, Serrano J, Del Valle E, Rodriguez M, Muñoz-Calero A (2014). Post-chemoradiation intraoperative electron-beam radiation therapy boost in resected locally advanced rectal cancer: long-term results focused on topographic pattern of locoregional relapse. Radiother Oncol.

[13] Liu B, Ge L, Wang J, Chen Y-Q, Ma S-X, Ma P-L (2021). Efficacy and safety of intraoperative radiotherapy in rectal cancer: a systematic review and meta-analysis. World J Gastrointest Oncol.

[14] Fahy MR, Kelly ME, Power Foley M, Nugent TS, Shields CJ, Winter DC (2021). The role of intraoperative radiotherapy in advanced rectal cancer: a meta-analysis. Colorectal Dis.

[15] Holman FA, Haddock MG, Gunderson LL, Kusters M, Nieuwenhuijzen GAP, van den Berg HA (2016). Results of intraoperative electron beam radiotherapy containing multimodality treatment for locally unresectable T4 rectal cancer: a pooled analysis of the Mayo Clinic Rochester and Catharina Hospital Eindhoven. J Gastrointest Oncol.

[16] Holman FA, Bosman SJ, Haddock MG, Gunderson LL, Kusters M, Nieuwenhuijzen GAP (2017). Results of a pooled analysis of IOERT containing multimodality treatment for locally recurrent rectal cancer: results of 565 patients of two major treatment centres. Eur J Surg Oncol.

[17] Calvo FA, Sole CV, Rutten HJ, Poortmans P, Asencio JM, Serrano J (2020). ESTRO/ACROP IORT recommendations for intraoperative radiation therapy in primary locally advanced rectal cancer. Clin Transl Radiat Oncol.

[18] Calvo FA, Sole CV, Rutten HJ, Dries WJ, Lozano MA, Cambeiro M (2020). ESTRO/ACROP IORT recommendations for intraoperative radiation therapy in locally recurrent rectal cancer. Clin Transl Radiat Oncol.

[19] Khullar K, Patel NM, Anderson C, Chundury A, Carpizo D, Feingold D (2020). The evolving role of radiotherapy in locally advanced rectal cancer and the potential for nonoperative management. Oncol Hematol Rev.

[20] Fu S, Lu JJ, Zhang Q, Yang Z, Peng L, Xiong F (2008). Intraoperative radiotherapy combined with adjuvant chemoradiotherapy for locally advanced gastric adenocarcinoma. Int J Radiat Oncol Biol Phys.

[21] Calvo FA, Sole CV, Obregón R, Gómez-Espí M, González-San Segundo C, González-Bayón L (2013). Intraoperative radiotherapy for the treatment of resectable locally advanced gastric adenocarcinoma: topography of locoregional recurrences and long-term outcomes. Clin Transl Oncol.

[22] Weese JL, Harbison SP, Stiller GD, Henry DH, Fisher SA (2000). Neoadjuvant chemotherapy, radical resection with intraoperative radiation therapy (IORT): improved treatment for gastric adenocarcinoma. Surgery.

[23] Miller RC, Haddock MG, Gunderson LL, Donohue JH, Trastek VF, Alberts SR (2006). Intraoperative radiotherapy for treatment of locally advanced and recurrent esophageal and gastric adenocarcinomas. Dis Esophagus.

[24] Zhang Q, Tey J, Peng L, Yang Z, Xiong F, Jiang R (2012). Adjuvant chemoradiotherapy with or without intraoperative radiotherapy for the treatment of resectable locally advanced gastric adenocarcinoma. Radiother Oncol.

[25] Drognitz O, Henne K, Weissenberger C, Bruggmoser G, Göbel H, Hopt UT (2008). Long-term results after intraoperative radiation therapy for gastric cancer. Int J Radiat Oncol Biol Phys.

[26] Hosokawa M, Shirato H, Ohara M, Kagei K, Hashimoto S, Nishino S (1999). Intraoperative radiation therapy to the upper mediastinum and nerve-sparing three-field lymphadenectomy followed by external beam radiotherapy for patients with thoracic esophageal carcinoma. Cancer.

[27] Murakami M, Kuroda Y, Nakajima T, Okamoto Y, Mizowaki T, Kusumi F (1999). Intraoperative radiotherapy for the abdominal lymphatic system in patients with esophageal carcinoma. Dis Esophagus.

[28] Tamaki Y, Sasaki R, Ejima Y, Ogura M, Negoro Y, Nakajima T (2012). Efficacy of intraoperative radiotherapy targeted to the abdominal lymph node area in patients with esophageal carcinoma. J Radiat Res.

[29] Gao P, Tsai C, Yang Y, Xu Y, Zhang C, Zhang C (2017). Intraoperative radiotherapy in gastric and esophageal cancer: meta-analysis of long-term outcomes and complications. Minerva Med.

[30] Yu W-W, Guo Y-M, Zhang Q, Fu S (2015). Benefits from adjuvant intraoperative radiotherapy treatment for gastric cancer: a meta-analysis. Mol Clin Oncol.

[31] Calvo FA, Sole CV, Obregón R, Gómez-Espí M, Lozano MA, Gonzalez-Bayon L (2013). Postchemoradiation resected locally advanced esophageal and gastroesophageal junction carcinoma: long-term outcome with or without intraoperative radiotherapy. Ann Surg Oncol.

[32] Cohen DJ, Leichman L (2015). Controversies in the treatment of local and locally advanced gastric and esophageal cancers. J Clin Oncol.

[33] Cuesta MA, van der Wielen N, Straatman J, van der Peet DL (2016). Video-assisted thoracoscopic esophagectomy: keynote lecture. Gen Thorac Cardiovasc Surg.

[34] Casas MA, Angeramo CA, Bras Harriott C, Schlottmann F (2021). Surgical outcomes after totally minimally invasive Ivor Lewis esophagectomy. A systematic review and meta-analysis. Eur J Surg Oncol.

[35] Biere SSY, Cuesta MA, van der Peet DL (2009). Minimally invasive versus open esophagectomy for cancer: a systematic review and meta-analysis. Minerva Chir.

[36] Straatman J, van der Wielen N, Cuesta MA, Daams F, Roig Garcia J, Bonavina L (2017). Minimally invasive versus open esophageal resection: three-year follow-up of the previously reported randomized controlled trial: the TIME trial. Ann Surg.

[37] Müller-Stich BP, Probst P, Nienhüser H, Fazeli S, Senft J, Kalkum E (2021). Meta-analysis of randomized controlled trials and individual patient data comparing minimally invasive with open oesophagectomy for cancer. Br J Surg.

[38] Cuesta MA, van der Wielen N, Straatman J, van der Peet DL (2017). Mastering minimally invasive esophagectomy requires a mentor; experience of a personal mentorship. Ann Med Surg (Lond).

[39] Scarcella S, Castellani D, Gauhar V, Teoh JY-C, Giulioni C, Piazza P (2021). Robotic-assisted versus open simple prostatectomy: results from a systematic review and meta-analysis of comparative studies. Investig Clin Urol..

[40] Hopland OA, Fosså SD, Ottosson F, Brennhovd B, Svindland A, Hole KH (2021). Robotic salvage pelvic lymph node dissection for locoregional recurrence after radical prostatectomy: a single institution experience. Scand J Urol.

[41] Tang B, Gao GM, Zou Z, Liu DN, Tang C, Jiang QG (2020). Efficacy comparison between robot-assisted and laparoscopic surgery for mid-low rectal cancer: a prospective randomized controlled trial. Zhonghua Wei Chang Wai Ke Za Zhi.

[42] Williams M, Perera M, Nouhaud FX, Coughlin G (2021). Robotic pelvic exenteration and extended pelvic resections for locally advanced or synchronous rectal and urological malignancy. Investig Clin Urol.

[43] Angeramo CA, Bras Harriott C, Casas MA, Schlottmann F (2021). Minimally invasive Ivor Lewis esophagectomy: robot-assisted versus laparoscopic-thoracoscopic technique. Syst Rev Meta-Anal Surg.

[44] Manigrasso M, Vertaldi S, Marello A, Antoniou SA, Francis NK, De Palma GD (2021). Robotic esophagectomy. A systematic review with meta-analysis of clinical outcomes. J Pers Med.

[45] Cerfolio RJ, Wei B, Hawn MT, Minnich DJ (2016). Robotic esophagectomy for cancer: early results and lessons learned. Semin Thorac Cardiovasc Surg.

[46] Wilson JD, Hammond EM, Higgins GS, Petersson K (2019). Ultra-high dose rate (FLASH) radiotherapy: silver bullet or fool’s gold?. Front Oncol.

[47] Gunderson LL, Willett CG, Harrison L, Calvo FA (2011). Intraoperative irradiation: techniques and results.

[48] Barnes M, Pass H, DeLuca A (1987). Response of the mediastinal and thoracic viscera of the dog to intraoperative radiation therapy (IORT). Int J Radiat Oncol Biol Phys.

[49] Tochner ZA, Pass HI, Sindelar WF, DeLuca AM, Grisell DL, Bacher JD (1992). Long term tolerance of thoracic organs to intraoperative radiotherapy. Int J Radiat Oncol Biol Phys.

[50] Powers BE, Gillette EL, McChesney SL, LeCouteur RA, Withrow SJ (1989). Bone necrosis and tumor induction following experimental intraoperative irradiation. Int J Radiat Oncol Biol Phys.

[51] LeCouteur RA, Gillette EL, Powers BE, Child G, McChesney SL, Ingram JT (1989). Peripheral neuropathies following experimental intraoperative radiation therapy (IORT). Int J Radiat Oncol Biol Phys.

[52] Gillette EL, Powers BE, McChesney SL, Withrow SJ (1988). Aortic wall injury following intraoperative irradiation. Int J Radiat Oncol Biol Phys.

[53] Kinsella TJ, Sindelar WF, DeLuca AM, Pezeshkpour G, Smith R, Maher M (1985). Tolerance of peripheral nerve to intraoperative radiotherapy (IORT): clinical and experimental studies. Int J Radiat Oncol Biol Phys.

[54] Schüler E, Acharya M, Montay-Gruel P, Loo BW, Vozenin M-C, Maxim PG (2022). Ultra-high dose rate electron beams and the FLASH effect: from preclinical evidence to a new radiotherapy paradigm. Med Phys.

[55] Monden K, Sadamori H, Hioki M, Ohno S, Takakura N (2022). Short-term outcomes of laparoscopic versus open liver resection for hepatocellular carcinoma in older patients: a propensity score matching analysis. BMC Surg.

[56] Bellantone R, Bossola M, Merrick HW, Doglietto GB, Ratto C, Minimo C (1992). Whole liver intraoperative irradiation after partial hepatectomy produces minimal functional and pathologic lesions. J Surg Oncol.

[57] Eisenberg BL, Lanciano RM, Nussbaum ML, Klein-Szanto A, Taylor DD (1992). Intraoperative liver radiation after partial hepatectomy in a rat model. J Surg Res.

[58] Kaiser GM, Mueller AB, Sauerwein W, Zhang HW, Westermann S, Frühauf NR (2005). Biliodigestive anastomosis after intraoperative irradiation in swine. J Invest Surg.

[59] Antoch G, Kaiser GM, Mueller AB, Metz KA, Zhang H, Kuehl H (2004). Intraoperative radiation therapy in liver tissue in a pig model: monitoring with dual-modality PET/CT. Radiology.

[60] Krengli M, Terrone C, Ballarè A, Loi G, Tarabuzzi R, Marchioro G (2010). Intraoperative radiotherapy during radical prostatectomy for locally advanced prostate cancer: technical and dosimetric aspects. Int J Radiat Oncol Biol Phys.

[61] Rocco B, Jereczek-Fossa BA, Matei D-V, Verweij F, Santoro L, Vavassori A (2009). Intraoperative radiotherapy during radical prostatectomy for intermediate-risk to locally advanced prostate cancer: treatment technique and evaluation of perioperative and functional outcome vs standard radical prostatectomy, in a matched-pair analysis. BJU Int.

[62] van Kampen M, Eble MJ, Krempien R, Jensen K, Aydenitz B, Metzger M (2003). Influence of irradiated volume on ureteral injury after intraoperative radiation therapy: experimental study in dogs. Radiology.

[63] DeLuca AM, Johnstone PA, Ollayos CW, Bacher JD, Terrill RE, Kinsella TJ (1994). Tolerance of the bladder to intraoperative radiation in a canine model: a five-year follow-up. Int J Radiat Oncol Biol Phys.

